# A comparative study of diagnostic accuracy in 3026 pleural biopsies and matched pleural effusion cytology with clinical correlation: A methodological issue

**DOI:** 10.1002/cam4.5525

**Published:** 2022-12-19

**Authors:** Wen‐Li Zhang, Xiang Fang, Jian‐Qing Qiu, Tao Cui

**Affiliations:** ^1^ Department of Orthopedics West China Hospital, Sichuan University Chengdu People's Republic of China; ^2^ West China Biomedical Big Data Center West China Hospital, Sichuan University Chengdu People's Republic of China; ^3^ Department of Gynecology and Obstetrics West China Second University Hospital, Sichuan University Chengdu People's Republic of China; ^4^ Key Laboratory of Birth Defects and Related Diseases of Women and Children (Sichuan University) Ministry of Education Chengdu People's Republic of China

## Abstract

**Aim:**

After reading the article by Ivan K. Poon et al. Certain issues regarding the methodology must be addressed.

**Result:**

First, it was not sufficient using only sensitivity and specificity. Second, we think the receiver operator characteristic curve (ROC) as well as the index of area under curve (AUC) could better be demonstrated in this, it was not sufficient using only sensitivity and specificity. Second, we think the receiver operator characteristic curve (ROC) as well as the index of area under curve (AUC) could better be demonstrated in this article for a more comprehensive understanding of this diagnostic test accuracy when comparing different tests.

**Conclusion:**

With using more comprehensive indexes including Youden's index, LR and AUC, the conclusions of Ivan K. Poon and his colleagues would be sodified.

## Graphical abstract

The comparison of SP and SE in various methods using a fixed cut‐point was affected by point selection. So we proposed the utilization of AUC for displaying the overall capabilities of those methods regardless of cut‐points.

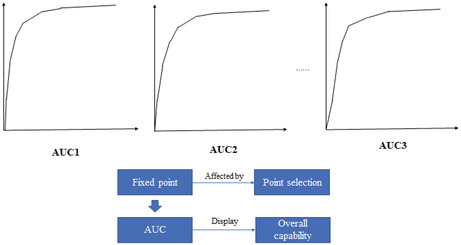

With great interest we read the article by Ivan K. Poon et al.^1^ In their study, they compared effusion cytology with pleural biopsies for malignant pleural effusion (MPE) accuracy evaluation. In this retrospective study, when taking account into B2/C2 diagnoses as negative and B5/C5 as positive, the specificity (SP), sensitivity (SN), positive predictive values (PPVs), negative predictive values (NPVs), and accuracies were 0.998, 0.495, 0.994, 0.742, and 0.782 for biopsy, 0.998, 0.570, 0.996, 0.791, and 0.835 for cytology. When considering B4/C4 diagnoses as positive, the SP, SN, PPVs, NPVs, and accuracies were 0.996, 0.517, 0.991, 0.724, and 0.786 for biopsy and 0.997, 0.634, 0.993, 0.791, and 0.845 for cytology. As a conclusion, the effusion cytology might be more reliable.

However, we would like to highlight two limitations. First, the authors only reported the SE, SP, PPVs, and NPVs, but those indexes were not sufficient for diagnostic accuracy evaluation. In order to better interpret the diagnostic accuracy from different perspective, more comprehensive indexes including diagnostic odds ratio, negative‐to‐positive likelihood ratio, as well as area under receiver operating characteristic curves (AUC) should be taken into account. As diagnostic accuracy is always defined as (true positives + true negatives)/(true positives + false negatives + false positives + true negatives), combing with two equally necessary perspectives: SE and SP, only report of single SE or SP was not helpful for understanding the overall diagnostic accuracy of the method. Moreover, the demonstration of receiver operating characteristic curve (ROC curve) and area under curves (AUC) was also necessary. On one hand, the ROC would tell the readers different SE and SP under different cut‐points for understanding the absolute diagnostic accuracy of the test. On other hand, only by comparing AUCs of different tests, the diagnostic added value of the new technology regardless of cut‐points would be yielded. As some accuracy measures can be admissible, while the diagnostic added values ultimately might be clinically negligible.^2^

In conclusion, with using more comprehensive indexes combing with ROC curve and AUC, the conclusions of the study by Ivan K. Poon and his colleagues would be considerably solidified.

## AUTHOR CONTRIBUTIONS

**Wenli Zhang:** Conceptualization (supporting); writing – original draft (lead). **Xiang Fang:** Writing – original draft (supporting). **Jianqing Qiu:** Writing – review and editing (equal). **Tao Cui:** Conceptualization (lead).

## FUNDING INFORMATION

None declared.

## CONFLICT OF INTEREST

The authors declare that they have no known competing financial interests or personal relationships that could have appeared to influence the work reported in this paper.

## Data Availability

Data sharing is not applicable to this article as no new data were created or analyzed in this study.
